# Dr. Westcott’s Introductory Lecture

**Published:** 1847-03

**Authors:** 


### INTRODUCTORY LECTURE BEFORE THE BALTIMORE COLLEGE OF DENTAL SURGEONS. BY DR. WESTCOTT.

It affords us pleasure to take notice of a lecture of this kind, in connection with those of the general profession. The ignorance and neglect of physicians and surgeons first made the diseases of the teeth a specialty, and created a class of operators as little acquainted with the principles of our science, as physicians and surgeons were with the manipulations of dentistry. Having been made a special branch, it is likely to continue such; and the question now is, shall it be rescued from the hands of pretenders and mere artists? We go in for its rescue, and shall at all times lend a hand towards its elevation. Having had some experience in both dental and ophthalmic surgery, we have no hesitation in saying, that the duties of the former require an equal knowledge of science, and greater dexterity of the hand, than those of the former; and still, while the scientific oculist is admitted to full rank in our profession, the scientific dentist is shut out. This is abundantly absurd, and we hope the time is not distant, when the learning, science and elevated bearing of those who devote themselves to that specialty, will redeem it from its congenital degradation. To this end, the dental colleges of Baltimore and Cincinnati cannot fail to contribute an abundant influence. Both have our best wishes.

The lecture of Dr. W., both in its manner and matter, will bear a comparison with the best introductories of the season; but we have extended our remarks so far as to have room for a single extract only:

"For some time, the scheme of establishihg a college expressly for this purpose was regarded by the community, and even by many of the profession, as chimerical. But thanks to the untiring exertions of its enterprising founder, the enlightened policy of the State Legislature, and to the cheering countenance and liberal support of the citizens of Baltimore, the experiment has been fully and successfully tried; the practicability and the great public utility of such an organization, have been fully proved; and the objections arising from the fears of friends, or the ill-boding prognostications of enemies have been most triumphantly refuted. This experiment has not only been successful in itself considered, but it has become the corner-stone of a new and more enlightened policy, both in regard to the public and the profession."

